# Tools for functional dissection of site-specific O-GlcNAcylation

**DOI:** 10.1039/d0cb00052c

**Published:** 2020-06-12

**Authors:** Andrii Gorelik, Daan M. F. van Aalten

**Affiliations:** Centre for Gene Regulation and Expression, School of Life Sciences, University of Dundee Dundee UK dmfvanaalten@dundee.ac.uk; Institute for Molecular Precision Medicine, Xiangya Hospital, Central South University Changsha China

## Abstract

Protein O-GlcNAcylation is an abundant post-translational modification of intracellular proteins with the monosaccharide *N*-acetylglucosamine covalently tethered to serines and threonines. Modification of proteins with O-GlcNAc is required for metazoan embryo development and maintains cellular homeostasis through effects on transcription, signalling and stress response. While disruption of O-GlcNAc homeostasis can have detrimental impact on cell physiology and cause various diseases, little is known about the functions of individual O-GlcNAc sites. Most of the sites are modified sub-stoichiometrically which is a major challenge to the dissection of O-GlcNAc function. Here, we discuss the application, advantages and limitations of the currently available tools and technologies utilised to dissect the function of O-GlcNAc on individual proteins and sites *in vitro* and *in vivo*. Additionally, we provide a perspective on future developments required to decipher the protein- and site-specific roles of this essential sugar modification.

## Abstract 2

An overview of the latest tools and technologies to investigate the roles of site-specific O-GlcNAcylation *in vitro* and *in vivo*.

## Graphical abstract

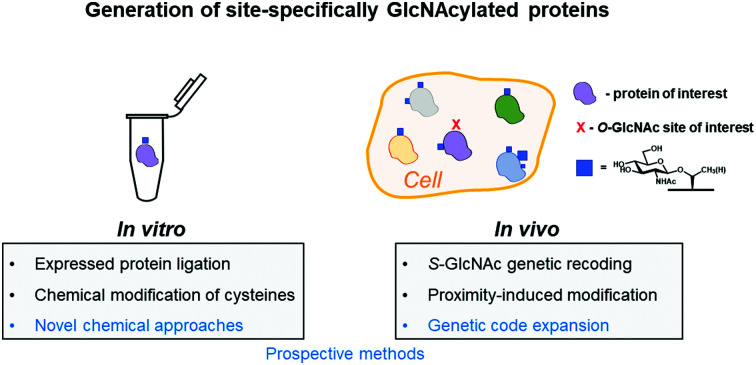

## Post(co)-translational protein O-GlcNAcylation

Post-translational modifications (PTMs) expand the function and regulation of proteins beyond the genetically encoded polypeptide. The number of known types of PTMs is close to several hundred,^1^ ranging from substantial alterations (proteolytic cleavage resulting in large protein fragments, attachment of ubiquitin chains, glycosylation with polysaccharides)^2–4^ to small tags (phosphorylation, acetylation, methylation, lipidation).^5–8^

**Fig. 1:**
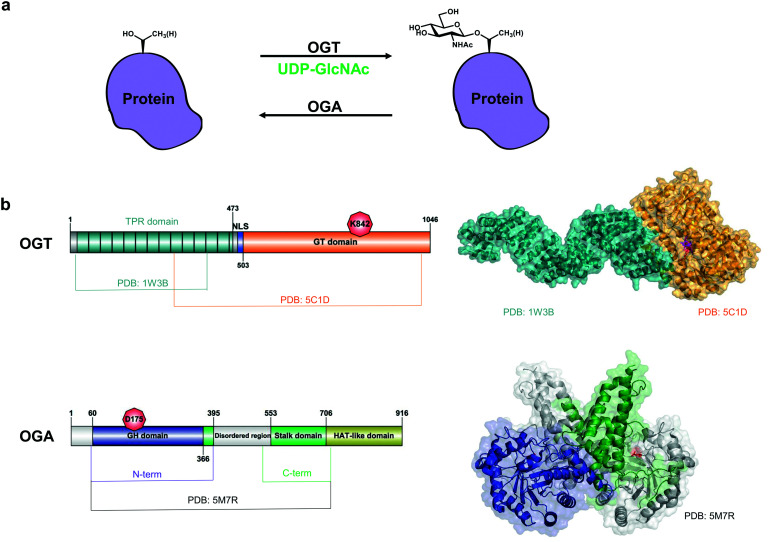
O-GlcNAc cycling on nucleocytoplasmic proteins. (a) OGT and OGA control the addition and removal of GlcNAc on serine and threonine residues. (b) Domain architecture and structures of OGT (composite of PDB: 1W3B and PDB: 5C1D) and OGA (PDB: 5M7R, lacks the disordered region). Catalytic site residues K842 (OGT) and D175 (OGA) are coloured in red. GT domain – glycosyltransferase domain. NLS – nuclear localization sequence,^40^ GH domain – glycoside hydrolase domain.

One of these PTMs, O-GlcNAcylation, is the attachment of a single O-linked *N*-acetylglucosamine onto serine and threonine side chains of intracellular proteins,^9^ which, in some cases, is also thought to be co-translational.^10^ Installation of the sugar is mediated by the O-GlcNAc transferase (OGT)^11,12^ on nuclear and cytoplasmic proteins, covering 10–20% of the whole human proteome (Fig. 1a).^13^ The O-GlcNAcase (OGA) opposes OGT by hydrolysing the O-glycosydic bond to release GlcNAc.^14^ Thus, a single pair of enzymes makes O-GlcNAc a reversible and highly dynamic modification that has been implicated in a myriad of processes such as signalling, metabolism, stress response and transcription.^15^

OGT is a family GT41 GT-B glycosyltransferase^16^ that consists of a 13.5 tetratricopeptide repeat (TPR) domain and a Rossman-like catalytic domain (Fig. 1b).^17^ Although the 13.5-TPR OGT is the major isoform, two additional isoforms may arise from alternative splicing and alternative start codons: a truncated OGT with 2.5 TPRs localised in the nucleus and cytoplasm and a 9.5-TPR OGT containing a mitochondrial targeting sequence.^18,19^ The function of the latter two isoforms is unclear. Moreover, it appears that full-length nucleocytoplasmic OGT is sufficient for O-GlcNAcylation of mitochondrial proteins.^20^ O-GlcNAc transfer by OGT involves an ordered bi-bi catalytic mechanism: binding of the donor sugar nucleotide UDP-GlcNAc is followed by the target protein acceptor substrate.^17,21^ A second catalytic activity of OGT, host cell factor-1 (HCF-1) cleavage, is performed in the same active site and results in proteolytic maturation of this transcriptional co-regulator.^22^ More recently, OGT was shown to possess a third (and unexpected) activity – catalysis of S-GlcNAc transfer onto cysteines.^23,24^ Although the crystal structures of the TPRs and the truncated OGT have been solved (Fig. 1b),^17,25^ the full-length OGT structure is yet to be determined and may provide a further understanding of how OGT recognizes its substrates. The TPR domain of OGT mediates substrate recognition through a so-called asparagine ladder.^17^ Using protein microarrays and mutational analysis, the five asparagines in the lumen of the TPR domain have been shown to contribute to the binding of OGT protein substrates beyond its active site.^26,27^ Recently, additional aspartates in the TPR domain that influence recognition of some OGT substrates have been identified.^28^ OGT does not utilise a defined motif for O-GlcNAcylation. However, in a high-throughput screen a preferred O-GlcNAc sequon has been determined as T-P-V-**gS/T**-R-A^12^ which is in good agreement with P-X-**gT**-X-A and P-V-**gS**^29^ and P/V-P/V-V-**gS/T**-S/T,^30,31^ previously identified in large-scale proteomics studies.

OGA is a GH84 family glycoside hydrolase^16^ consisting of an N-terminal catalytic domain and a histone acetyltransferase (HAT)-like domain separated by a disordered region and a stalk domain (Fig. 1b). The HAT-like domain lacks the ability to bind acetyl coenzyme A (the substrate for acetylation) and its role remains enigmatic.^32^ The putative short human OGA isoform lacks the HAT-like domain and a portion of the stalk domain, resulting in reduced activity due to the inability to form a functional dimer.^33^ Overexpression of this isoform with a GFP tag results in its localisation to lipid droplets.^34^ However, the short OGA isoform has not been detected at an endogenous protein level. The first crystal structures of a monomeric bacterial orthologue of human OGA from *Clostridium perfringens* (*Cp*OGA) in complex with its substrate peptides exhibited a V-shaped peptide conformation in the active site.^35,36^ Later it was shown that human OGA forms a dimer with a substrate-binding cleft where O-GlcNAc peptides assume the same V-shaped conformation upon binding in the active site, confirming the findings obtained with *Cp*OGA.^33,37,38^ While the interactions of OGA active site residues with GlcNAc are conserved, exactly how (and whether) protein substrates bind beyond the active site is unknown.^39^ OGA itself contains a single O-GlcNAcylation site at Ser405, which may regulate OGA stability.^24^

The roles of O-GlcNAc on proteins have been studied in animal models (such as mice and fruit flies), where OGT deletion is lethal and causes severe developmental abnormalities.^41–43^ Excess of O-GlcNAc can also have negative impact on normal cell physiology as OGA knockout is lethal in mice,^44^ whereas increased O-GlcNAc levels have been linked to oncogenic reprogramming.^45^ Since its discovery, the relationship between O-GlcNAcylation and phosphorylation has been closely studied due to the frequent occurrence on the same or adjacent Ser/Thr residues which results in mutual regulation.^46^ The role of O-GlcNAc in stress response is exemplified by its participation in stress granule formation and oxidative stress.^47^ At individual protein level, lack of O-GlcNAc on tau and α-synuclein has been associated with pathological brain conditions such as Alzheimer's and Parkinson's disease.^48,49^ Moreover, O-GlcNAc can mediate transcription through modification of RNA Polymerase II^50^ and regulate autophagosome maturation by modifying SNAP29 depending on the cellular metabolic state.^51^

Transcriptionally, OGT expression can be decreased through nuclear degradation of an intron-retained OGT transcript in response to high overall O-GlcNAcylation levels to maintain O-GlcNAc homeostasis.^52^ Similar to OGT, OGA expression is also responsive to the modulation of overall O-GlcNAc modification. Pharmacological inhibition of OGA in cells increases total O-GlcNAcylation levels, while also increasing OGA expression and decreasing expression of OGT as a compensatory mechanism.^53^

Recently, *ogt* missense mutations associated with X-linked intellectual disability have been identified.^54^ These occur in the TPR domain of OGT^54–56^ as well as in the catalytic domain.^57,58^ Some of these mutations cause a decrease in endogenous OGT activity.^55,57^ Therefore, it is possible that abolished O-GlcNAcylation status on a subset of OGT substrates or even a single substrate/site could contribute to the intellectual disability phenotype, an avenue that is currently being explored in our laboratory using several methods discussed in this review.

Currently, the O-GlcNAc field is experiencing rapid growth with researchers from related disciplines discovering that O-GlcNAc modulates the function of their proteins of interest. Examples include discoveries of the role of O-GlcNAcylation in preventing the aggregation of a Polycomb member, polyhomeotic, in *Drosophila*,^59^ as well as in controlling the anti-inflammatory function on receptor-interacting serine/threonine-protein kinase 3 (RIPK3)^60^ and in activating phosphoglycerate kinase 1 (PGK1) to promote glycolysis,^61^ to name a few. As the roles of O-GlcNAc are becoming recognized, there is an emerging need for tools to dissect site-specific O-GlcNAcylation.

### Visualisation of protein- and site-specific O-GlcNAcylation

A crucial step in studying any PTM is its detection. Although mass spectrometry (MS) provides a high-throughput method for O-GlcNAc identification and site-mapping (reviewed elsewhere^62^), it is complicated by time-consuming sample preparation, expensive equipment and (often difficult) analysis. Therefore, the use of gel and western blot methods is generally required which will be discussed below.

In the early days of the field, O-GlcNAc was detected by galactosyltransferase labelling with an [^3^H]-galactose followed by autoradiography and by lectin binding (for instance, with fluorescently labelled wheat germ agglutinin, which binds terminal GlcNAc residues).^63,64^ This was later surpassed by a superior chemoenzymatic method based on the mutant β-1,4-galactosyltransferase (GalT^Y289L^) that transfers an azide derivative of GalNAc (GalNAz) onto O-GlcNAcylated proteins.^65^ The azide provides a reactive handle for the attachment of tags using copper-catalysed azide–alkyne cycloaddition (CuAAC) or copper-free strain-promoted azide–alkyne cycloaddition (SPAAC),^66,67^ allowing qualitative and quantitative detection of O-GlcNAc.^66,68^ The use of mass tags (such as polyethylene glycols PEG5000- or PEG2000-alkyne) allows resolution of GlcNAc-modified proteins on a gel and determination of absolute stoichiometry using a western blot with an antibody against the target protein (eliminating the need for pulldowns and O-GlcNAc-specific antibodies).^66^ Additionally, the number of bands shifted indicates the number of O-GlcNAc sites. Unfortunately, the detection limit of the PEGylation method is approximately 5% (depending on the quality of the antibody) and the labelling efficiency relies on full completion of the galactosyltransferase reaction.^66^ The efficiency of the subsequent chemical addition of a PEG mass tag may also vary depending on the reagent concentration. To address this issue, the SPAAC reaction has been optimised using a synthetic protein standard (bearing stoichiometric O-GlcNAc modification) and commercial reagents.^69^ As an alternative to mass tagging, a native polyacrylamide gel electrophoresis (native PAGE) method based on co-polymerised *Cp*OGA has been developed in our laboratory (C. Fu and D. M. F. van Aalten, *Analyst*, in press). The retardation of an O-GlcNAc-modified band happens as a result of high-affinity binding by the catalytically inactive *Cp*OGA. Conveniently, this approach does not require any chemical modification of the sample.

Sometimes the stoichiometry of O-GlcNAc on a given protein is below the detection limit of antibodies. In order to amplify the O-GlcNAc signal in such cases, a proximity-ligation based method has been developed. First, O-GlcNAc is chemoenzymatically labelled with GalNAz, followed by a reaction with an alkyne-biotin. The next step is the coupling of antibody-DNA conjugates targeted to either biotin or the protein of interest. If the protein is O-GlcNAcylated, the antibodies are brought into proximity, leading to ligation of DNA tags. qPCR is subsequently performed to quantify the signal.^70^ While this method allows detection of O-GlcNAcylated proteins in small amounts of sample, the specificity of O-GlcNAc signal heavily relies on the specificity of an antibody against a protein of interest which could otherwise detect off-targets.

The donor substrate promiscuity of OGT has been extensively exploited to generate various metabolic reporters for global O-GlcNAc profiling by mass-spectrometry and in-gel visualisation. These modified sugars can be fed to cells where they enter specific metabolic pathways and can be utilised by OGT as donor substrates. These include GlcNAc derivatives with alkyne and azide functionalities such as GlcNAz,^71^ GlcNAlk,^72^ 6AzGlcNAc,^73^ 4-deoxy-GlcNAz,^74^ 2AzGlc,^75^ 6AlkGlcNAc,^76^ 6AzGlc^77^ and GlcNDAz.^78^

Antibodies remain the most popular tool to detect O-GlcNAc. The two most widely used pan-specific O-GlcNAc antibodies are RL2 (originally developed for detecting nuclear pore proteins)^64,79^ and CTD110.6 (developed based on the O-GlcNAcylated RNA polymerase II C-terminal domain).^80^ Although both of these commercially available antibodies are monoclonal, RL2 exclusively binds to O-GlcNAc, while CTD110.6 may also recognise *N*-GlcNAc, GlcNAcylated O-mannose, cross-reacts with terminal β-GlcNAc on complex *N*-glycans of cell surface glycoproteins and more recently has been shown to efficiently detect S-GlcNAc on cysteines.^24,81–84^ Therefore, care must be taken when using CTD110.6 antibody whose specificity must be tested in each case with an OGA-treated negative control. Although RL2 binds O-GlcNAc with greater specificity, it may not recognise O-GlcNAcylation in certain contexts such as demonstrated with α-synuclein stoichiometrically O-GlcNAc-modified at several reported O-GlcNAcylation sites.^85^

Unlike in the field of phosphorylation, very few site-specific O-GlcNAc antibodies have been generated. Nevertheless, these have greatly aided the study of site-specific O-GlcNAc modification and are summarised in Table 1. For instance, reciprocal interplay between O-GlcNAcylation and proximal phosphorylation could be examined using antibodies against O-GlcNAcylated Thr58 on c-Myc (first site-specific O-GlcNAc antibody ever produced)^86^ and O-GlcNAcylated Ser400 on tau.^46^ Our laboratory had generated a Ser395-O-GlcNAc-specific TGF-β activated kinase-1 (TAK1) binding protein-1 (TAB1) antibody that revealed increased O-GlcNAcylation as a modulator of TAK1 signalling upon IL-1 stimulation and osmotic stress.^87^ More recently, we produced a Ser517-O-GlcNAc-specific CRMP2 antibody to show an age-dependent increase in CRMP2 O-GlcNAcylation associated with short-term memory impairment.^88^

**Table 1:** Site-specific O-GlcNAc antibodies generated to date

O-GlcNAc antibody	Site (human)	Ref.
c-Myc	Thr58	86
Collapsin response mediator protein-2 (CRMP2)	Ser517	88
Histone H2A	Ser40	89
Histone H2A	Thr101	www.glycoscientific.com/
Histone H2B	Ser112	90
Histone H3	Thr32	www.glycoscientific.com/
Histone H4	Ser47	www.glycoscientific.com/
Insulin receptor substrate-1 (IRS1)	Ser1011	91
Insulin receptor substrate-2 (IRS2)	Thr1155	www.glycoscientific.com/
NAD-dependent protein deacetylase Sirtuin-1 (SIRT1)	Ser549	92
Tau	Ser400	93 and 94
TGF-beta-activated kinase 1-binding protein 1 (TAB1)	Ser395	87
TGF-beta-activated kinase 1-binding protein 3 (TAB3)	Ser408	95

### Enzymatic methods for generating protein-specific O-GlcNAcylation

**Fig. 2:**
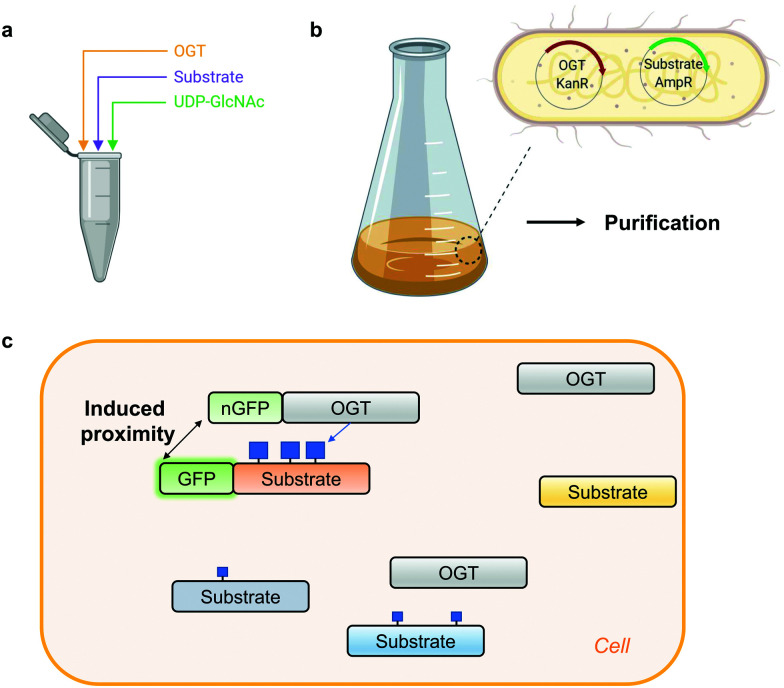
Methods for generating protein-specific O-GlcNAcylation. (a) *In vitro* OGT reaction. (b) Co-expression of OGT with its substrate in *E. coli*, followed by purification. KanR – kanamycin resistance, AmpR – ampicillin resistance. (c) Proximity OGT approach for protein-selective O-GlcNAcylation in cells.^100^ nGFP – anti-GFP nanobody. Blue squares denote O-GlcNAc.

Access to stoichiometrically modified proteins is often required to dissect the mechanistic consequences of PTMs *in vitro* and *in vivo*. However, producing highly O-GlcNAc-modified proteins is challenging. Traditionally, the simplest approach to obtain an O-GlcNAcylated protein of interest has been *in vitro* reaction with OGT (Fig. 2a).^96^ Despite the ease of implementation, to achieve high O-GlcNAc stoichiometry, long incubation times are generally needed which in itself may have undesired effects on the stability of the protein under investigation. Alternatively, to preserve protein stability, OGT can be co-expressed with its substrates in *E. coli* or insect cells (Fig. 2b). Application of this system in a bacterial setting has been demonstrated for the RNA-Polymerase II C-terminal domain, TAB1, calcium/calmodulin-dependent protein kinase type IV (CaMKIV), Tau, Coactivator Associated Arginine Methyltransferase 1 (CARM1) and nuclear pore glycoprotein p62 (nup62).^96–98^ However, an endogenous glycosidase in *E. coli* may compromise the yield of the O-GlcNAc-modified proteins produced with this approach.^99^ In insect (Sf9) cells simultaneous expression of CREB and OGT led to a three-fold increase of CREB glycosylation to almost 90%.^66^ Although this co-expression approach may not be compatible with all OGT substrates, the main advantage is the ability to produce co-translationally O-GlcNAc-modified proteins which is impossible *via* OGT reaction *in vitro*.

Recombinant O-GlcNAc-modified protein on its own is usually not sufficient to dissect the function of this modification. To acquire control over a single-protein O-GlcNAcylation status in a complex cellular environment, a creative way to induce proximity of OGT to its substrates in cells has been proposed recently (Fig. 2c).^100^ Exploiting the fusion of OGT to a nanobody against a tag (GFP or RFP) on a protein of interest or a nanobody against endogenous protein target, Woo and colleagues managed to increase O-GlcNAcylation stoichiometry on a subset of OGT substrates. In its current form this proximity induction relies on the overexpression of OGT which concomitantly elevates global O-GlcNAc levels. This strategy may have potential to increase O-GlcNAcylation on a protein of interest, if the impact of OGT overexpression on the global O-GlcNAcome can be addressed. Alternatively, other proximity approaches could be explored, such as the use of aptamers or small molecules (PROTAC principle). For example, the use of a heterobifunctional small molecule has already been utilised to bring protein phosphatase 1 (PP1) in proximity to its targets resulting in a protein-specific decrease in phosphorylation of AKT kinase and EGFR.^101^ One of the most important considerations for the future development of such probes is that the interaction of the proximity-inducing molecules does not affect the catalytic or scaffolding function of OGT or its substrate.

Current methods to increase protein-specific O-GlcNAcylation still result in heterogeneous mixtures of modified and unmodified proteins and make functional analysis particularly cumbersome in case of multiple glycosylation sites. Thus, knowledge of specific O-GlcNAc sites and application of approaches to generate site-specifically O-GlcNAcylated proteins could circumvent this issue.

### Chemical biology methods for dissecting site-specific O-GlcNAcylation *in vitro*

**Fig. 3:**
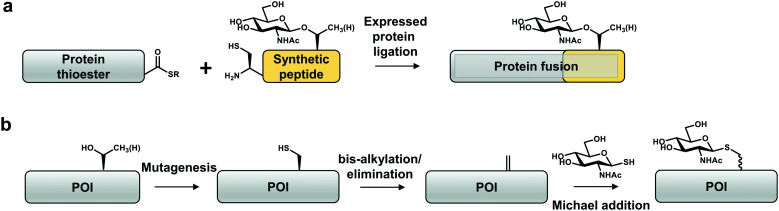
Site-specific chemical biology approaches to study O-GlcNAcylation *in vitro*. (a) Expressed protein ligation. (b) “Tag-and-modify” dehydroalanine approach. POI – protein of interest.

One approach that has been explored to incorporate site-specific O-GlcNAcylation is expressed protein ligation (EPL), a semi-synthetic strategy to obtain homogenous and stoichiometric site-specific PTMs of a target protein (Fig. 3a).^102,103^ EPL involves synthesis of a peptide bearing a desired functionality (*e.g.* post-translational modification), which is then ligated *via* S-to-*N* acyl-transfer to a thioester generated from a recombinant intein-fusion protein.^102^ To date, tau,^104^ α-synuclein,^49,85,105^ HSP27^106^ and an unnaturally modified ubiquitin^69^ are the only four reported O-GlcNAcylated proteins produced using EPL. This method has been used to dissect the mechanistic consequences of O-GlcNAcylation on α-synuclein (Thr72, Thr75, Thr81, Ser87),^49,85,105^ which is a small (<15 kDa) protein amenable to EPL. This approach revealed that O-GlcNAc on α-synuclein affects aggregation and toxicity *in vitro*.^85^ Apart from a handful of O-GlcNAcylated proteins, S-GlcNAcylated casein kinase 2 (CK2)^107^ and α-synuclein^108^ have been produced by EPL in order to increase the stability of the modification against hydrolysis. Through structural and biophysical studies it was shown that S-GlcNAc serves as a good non-hydrolysable analogue for O-GlcNAc that can be recognized by some O-GlcNAc-specific antibodies (Ser395-O-GlcNAc-TAB1 and CTD110.6).^24,108^

In future the utility of CHF or CF_2_ functionalized GlcNAc as potentially nonhydrolyzable analogues for EPL could be investigated as these have been shown to be good steric and electronic representations in the context of O-phosphorylation.^109^ The main disadvantage of EPL is that it must be optimised for every protein and the maximum length of the peptide for ligation is currently limited. Importantly, this method requires good expression of a soluble intein-fusion protein, which frequently poses a problem. Insertion of modified peptides into the central portions of large, globular proteins involves iterative ligation steps and is particularly challenging. A cysteine residue that EPL introduces at the site of ligation cannot always be converted to a native amino acid (*e.g.* to alanine *via* desulphurisation). Therefore, the site of ligation must be carefully considered.

In another approach, taking advantage of the potent nucleophilicity and low abundance of cysteine in proteins, cysteine-conversion chemical methods have been developed by Davis and co-workers to obtain proteins with site-specific O-GlcNAc mimics *in vitro* (Fig. 3b).^110–112^ These rely on the chemical transformation of a genetically installed cysteine into dehydroalanine by an alkylating reagent (for example, O-mesitylenesulfonylhydroxylamine (MSH) or 2,5-dibromohexanediamide (DBHDA)) under denaturing conditions. The dehydroalanine of the unfolded protein is then incubated with a reactive GlcNAc derivative (*e.g.* GlcNAc-thiol) and the modified protein is refolded.^110–112^ Notably, a thiol-linked GlcNAc prepared in such a way is resistant to reduction by DTT.^113^ Moreover, endoglycosidase-A exhibits trans-glycosylation activity for a synthetic S-linked GlcNAc (S-GlcNAc)-modified protein with a high modification efficiency.^112^ Cys-S-GlcNAcylation at position 101 (Thr in the native protein) of histone H2A was shown to destabilise H2A/H2B dimers, promoting an open chromatin state. Installation of S-GlcNAc at position 112 of histone H2B (Ser in the native protein) allowed identification of interactors, among which are subunits of the FACT chromatin remodelling complex.^110,111^ As evidence for physiological mimicry, O-GlcNAc-homohomo-Ser prepared by this approach can be hydrolysed by human OGA.^114^ This method is limited to recombinant proteins with no or few native cysteine residues, that otherwise must first be substituted by other amino acids (Ser or Ala). Furthermore, this approach does not allow control of stereo-specificity of the modification rendering it difficult to interpret the results of the experiment.

Another method for installing S-GlcNAc employs a thio-glycoligase capable of transferring GlcNAc to cysteines, engineered from a *Streptomyces plicatus* hexosaminidase *via* mutation of the catalytic glutamate (E314A).^115^ Withers and co-workers managed to produce S-GlcNAcylated synuclein peptides and tau protein using this new method.^115^ As with the dehydroalanine approach, this technique requires mutation of all native cysteines that are not destined for S-GlcNAc modification and its utility is limited to *in vitro* reactions.

### Genetic methods for dissecting site-specific O-GlcNAcylation *in vivo*

Loss-of-function mutations are the most common approach to probe site-specific O-GlcNAcylation in cultured cells (*e.g.* Ser/Thr mutation to Ala). For example, Ser529Ala mutation on phosphofructokinase 1 was shown to prevent O-GlcNAc-dependent increase in its activity,^116^ while Thr228Ala mutation of Oct4 reduces stem cell self-renewal and reprogramming.^117^ The caveat of this approach lies in the loss of the side chain which in itself could have an impact on protein folding and stability. Therefore, Ala mutagenesis is often used in combination with other methods to down- or upregulate OGT or OGA levels and their enzymatic activity. OGA knockout, knockdown and inhibition is an effective way to elevate total O-GlcNAc modification levels *in vivo* and in cell culture, since O-GlcNAcylation is often sub-stoichiometric in cells (<10%) due to the high OGA activity.^118–120^ However, with this approach, functional dissection of the roles of individual O-GlcNAc sites is impeded by potential ambiguous phenotypes through effects on many other OGT substrates.

**Fig. 4:**
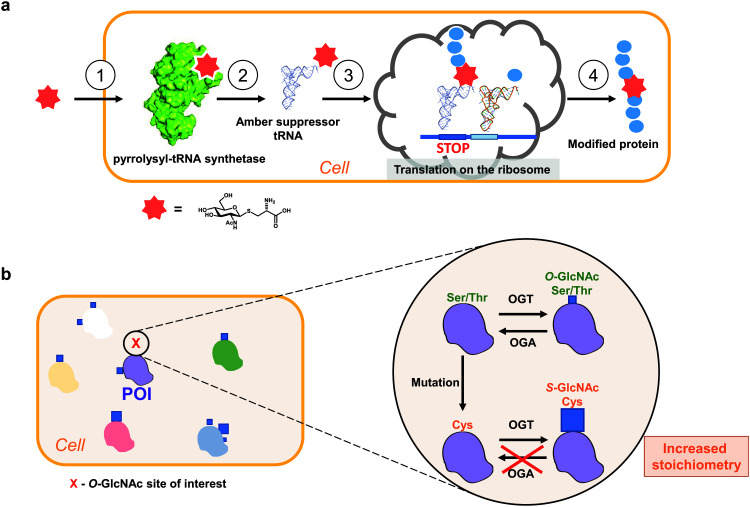
Approaches to study site-specific O-GlcNAcylation *in vivo.* (a) Principle of a genetic code expansion approach with a metabolically stable Cys-S-GlcNAc amino acid: (1). UAA uptake, (2). tRNA aminoacylation, (3). UAA translation in response to a stop codon, (4). Production of a modified protein. (b) Genetic recoding approach to introduce site-specific S-GlcNAc mimicry of O-GlcNAcylation.^24^ Blue squares denote GlcNAc. POI – protein of interest.

Genetic code expansion (GCE) technology allows site-specific incorporation of unnatural amino acids (including PTMs) *in vitro* and *in vivo* by utilising evolved orthogonal amber suppressor tRNA synthetases (Fig. 4a).^121–125^ The GCE method often requires evolution of an archaeal pyrrolysine-tRNA synthetase (PylRS) to aminoacylate a corresponding amber suppressor tRNA, which in turn allows decoding of the unnatural amino acid (UAA) of interest at an amber stop (or quadruplet^121^) codon in a genetically predetermined fashion (Fig. 4a). Since the amber codon is the least abundant of the three stop codons, its use reduces the off-target incorporation of the amino acid where endogenous amber stop codons occur.

Co-translational incorporation of a glycosylated amino acid by the GCE technology could become a promising tool to site-specifically examine the gain-of-function O-GlcNAc modification *in vivo*.^122–124^ With over 200 UAAs incorporated to date,^126^ the expanded genetic code includes several PTMs such as phosphorylated amino acids (serine, threonine and tyrosine), acetylated lysine and a scaffold for ubiquitin- and SUMO-modified lysine.^127–132^ Most of the UAAs incorporated by the PylRS-based GCE are lysine derivatives that resemble the natural substrate of PylRS, pyrrolysine.^126^ Amino acids as polar as GlcNAcylated serine or cysteine have not yet been added to the expanded genetic code. Apart from the specificity and activity of the evolved PylRS, incorporation of UAAs largely depends on the tolerance of the corresponding aminoacylated tRNA by the translation machinery, such as the ribosome, elongation factor Tu and release factors. These problems were previously encountered with phosphoserine, for which some components of the translational machinery had to be additionally engineered.^128^

A major obstacle in applying GCE to O-GlcNAc is the uptake and stability of the synthetic O-GlcNAcylated amino acid. *E. coli*, which is used as a host system for evolving PylRS, metabolises Ser-O-GlcNAc as a carbon source,^133^ precluding the use of this UAA for GCE.^134^ De-acetylation of a per-O-acetylated Ser-O-GlcNAc variant (which would hypothetically increase the cellular uptake) does not occur in *E. coli* as it does not express the requisite deacetylases.^133^ The latter strategy would be more suited to mammalian cells, where pan-specific de-acetylating enzymes do exist. Importantly, however, we showed that a synthetic amino acid Cys-S-GlcNAc, a Ser-O-GlcNAc analogue, is efficiently internalised by *E. coli* where it remains metabolically stable, satisfying the first essential step and prerequisite for directed evolution of PylRS.^135^

Recombinant proteins with a glycosylation mimic have been obtained indirectly through PylRS amber suppression, by introducing an amino acid with the alkene functionality followed by a click chemistry reaction.^136^ Recently, cellular incorporation of dehydroalanine was demonstrated.^137^ This allowed the authors to install thio-linked GlcNAc at a defined position on GFP which was recognised by a pan-specific O-GlcNAc antibody.^137^

Although glycosylated amino acids have not been incorporated by GCE, there are some clues as to the feasibility of this approach reported in several studies.^138,139^ In these reports glycosylated recombinant proteins were produced in a cell-free system using chemically aminoacylated amber suppressor tRNAs.^138,139^ While the efficiencies of amber suppression varied between different glycosylated amino acids reaching up to 30%,^138^ in one of the studies, it was shown that Thr-O-GalNAc could be incorporated only at the protein N-terminus, suggesting that glycosylated polypeptides are not well tolerated by the ribosome.^139^

The major advantage of GCE is the ability to produce stoichiometrically-modified proteins in living systems. On the other hand, the level of incorporation strongly depends on the suppression efficiency of the stop codon. This does not pose significant issues for purified recombinant proteins (where the expression can be scaled up). However, when interpreting the effects of a post-translational modification *in vivo*, it is challenging to provide appropriate controls (*i.e.* achieving the same expression as for the unmodified protein). Therefore, any observed effects resulting from incorporation of a UAA could also be the result of altered expression levels. Undesired off-target effects could also be observed in case of mis-incorporation of the amino acid in unintended proteins in place of endogenous stop codons, resulting in skewed phenotypes. Future developments of GCE are required to address these issues.

To gain insight into site-specific O-GlcNAcylation in cells, our laboratory has explored a surprisingly simple approach to genetically introduce a non-hydrolysable S-GlcNAc *in vitro* and *in vivo* using Ser/Thr to Cys mutagenesis and relying on the fortuitous promiscuity of OGT that happens to possess efficient S-GlcNAc transferase activity (Fig. 4b).^24^ Notably, this method does not require any exogenous biomolecular machinery, overexpression of (mutant) genes or chemical synthesis and can be combined with the CRISPR-Cas9 genome editing technology. With this approach, the stoichiometry of cysteine GlcNAcylation can replicate that of OGA inhibition in cells due to the hydrolytic stability of S-GlcNAc. However, unlike OGA inhibition, S-GlcNAc mutagenesis does not affect global O-GlcNAc levels, minimizing undesired indirect effects. By applying this method to a single Ser O-GlcNAcylation site on OGA and converting it to a Cys S-GlcNAcylation site, we managed to increase the stoichiometry almost five-fold (from 15% to over 70%), similar to that achieved with the OGA inhibitor treatment.^24^ We showed that the high GlcNAcylation stoichiometry decreased OGA stability in cells, while the overall thermal stability remained unchanged relative to the wild type protein.^24^ Interestingly, in the same sequence contexts S-GlcNAc appears to be more stable to CID MS/MS fragmentation^23^ and may assist in mapping and detection of O-GlcNAc sites. Additionally, homogenous site-specifically S-GlcNAcylated recombinant proteins can be produced through GlcNAcylation of a Cys mutant protein and subsequent treatment with OGA (for example, the highly-active *Cp*OGA) to remove unwanted O-GlcNAcylation (if multiple modification sites are present). It must be noted that in cases where OGA inhibition does not elevate O-GlcNAcylation levels on a protein of interest in cells, this approach may not be applicable. While this S-GlcNAc genetic recoding approach has been applied in cultured human and mouse cells, it has yet to be demonstrated that this leads to site-specific S-GlcNAc incorporation in animal models.

## Conclusions

The functional consequences of protein O-GlcNAcylation still remain poorly understood due to the limited number of tools to study its site-specific effects. As can be appreciated from this review, no single method to study protein- and site-specific O-GlcNAcylation can be universally applied and all of the discussed techniques have their advantages and disadvantages. Thus, a combination of these approaches can help us achieve a comprehensive understanding of the functions of the O-GlcNAc PTM on specific proteins and sites. The future developments in the field require optimization of contemporary methods and invention of new strategies to gain control of protein- and site-specific modification. Expanding the genetic code with glycosylated amino acids would represent just one of these future advances. Ingenious methods are required to achieve protein- and site-specific O-GlcNAcylation with minimal side effects and to avoid unnecessary non-physiological perturbation in living systems. The use of non-hydrolysable analogues of O-GlcNAc (such as S-, CHF- and CF_2_-linked GlcNAc or other analogues^24,74,140^) in an intracellular setting must be considered to withstand the high activity of OGA that could otherwise abolish the efforts of site-specific installation of O-GlcNAc. Spatiotemporal control of site-specific O-GlcNAcylation in cells is also important, since O-GlcNAc is a signalling molecule that can lead to rapid activation of signalling cascades. Last but not least, low O-GlcNAcylation stoichiometry at a given site can be the result of protein–protein interactions that “mask” such modification sites and prevent subsequent post-translational modification by OGT, thus requiring implementation of techniques for co-translational site-specific GlcNAc incorporation. Bridging the gap between chemistry and biology is required to invent approaches that fine-tune O-GlcNAc stoichiometry with a single protein and amino acid residue precision. Such interdisciplinary amalgamation will be instrumental in investigating the diverse functions of protein O-GlcNAcylation.

## Conflicts of interest

There are no conflicts of interest to declare.

## Supplementary Material
